# Supplementary material for: A fault-intrusion-tolerant system and deadline-aware algorithm for scheduling scientific workflow in the cloud
Source: PeerJ Comput Sci. 2021 Nov 2;7:e747. doi: 10.7717/peerj-cs.747 (PMC8576554; doi:10.7717/peerj-cs.747)
Supplement: Supplemental Information 6 [file peerj-cs-07-747-s006.zip › Myproject1.1/docs/allclasses-frame.html]

All Classes


# All Classes

- AbstractArrayList
- AggregateSwitch
- AppCloudlet
- ArgumentParser
- BalancedClustering
- BalancingMethod
- *BalancingMetric*
- BasePlanner
- BaseScheduler
- BasicClustering
- BlockClustering
- BwProvisioner
- BwProvisionerSimple
- ChildAwareHorizontalClustering
- CloudInformationService
- Cloudlet
- CloudletList
- CloudletScheduler
- CloudletSchedulerDynamicWorkload
- CloudletSchedulerSpaceShared
- CloudletSchedulerTimeShared
- CloudSim
- CloudSimExample1
- CloudSimExample2
- CloudSimExample3
- CloudSimExample4
- CloudSimExample5
- CloudSimExample6
- CloudSimExample7
- CloudSimExample8
- CloudSimExample8.GlobalBroker
- CloudSimShutdown
- CloudSimTags
- ClusteringEngine
- *ClusteringInterface*
- ClusteringParameters
- ClusteringParameters.ClusteringMethod
- ClusterStorage
- CondorVM
- Constants
- Consts
- *ContinuousDistribution*
- DataAwareScheduler
- Datacenter
- DatacenterBroker
- DatacenterCharacteristics
- DatacenterExtended
- DataCloudTags
- DeferredQueue
- DelayMatrix\_Float
- DistanceVariance
- Dvfs
- Dvfs
- EdgeSwitch
- ExecutionTimeMeasurer
- ExponentialDistr
- FailureGenerator
- FailureMonitor
- FailureRecord
- FCFSScheduler
- File
- FileAttribute
- FloydWarshall\_Float
- FutureQueue
- GammaDistr
- GraphReaderBrite
- *GraphReaderIF*
- HarddriveStorage
- Helper
- HorizontalClustering
- HorizontalDistanceBalancing
- HorizontalImpactBalancing
- HorizontalRandomClustering
- HorizontalRuntimeBalancing
- HorizontalRuntimeVariance
- Host
- HostDynamicWorkload
- HostList
- HostPacket
- HostStateHistoryEntry
- ImpactFactorVariance
- InfoPacket
- IqrMc
- IqrMc
- IqrMmt
- IqrMmt
- IqrMu
- IqrMu
- IqrRs
- IqrRs
- Job
- Log
- LognormalDistr
- LomaxDistribution
- LrMc
- LrMc
- LrMmt
- LrMmt
- LrMu
- LrMu
- LrrMc
- LrrMc
- LrrMmt
- LrrMmt
- LrrMu
- LrrMu
- LrrRs
- LrrRs
- LrRs
- LrRs
- MadMc
- MadMc
- MadMmt
- MadMmt
- MadMu
- MadMu
- MadRs
- MadRs
- MathUtil
- MaxMinScheduler
- MCTScheduler
- MinMinScheduler
- NetDatacenterBroker
- NetworkCloudlet
- NetworkCloudletSpaceSharedScheduler
- NetworkConstants
- NetworkDatacenter
- NetworkExample1
- NetworkExample2
- NetworkExample3
- NetworkExample4
- NetworkHost
- NetworkPacket
- NetworkTopology
- NetworkVm
- NetworkVmAllocationPolicy
- NonPowerAware
- NonPowerAware
- OverheadParameters
- *Packet*
- ParameterException
- Parameters
- Parameters.FTCFailure
- Parameters.FTCMethod
- Parameters.FTCMonitor
- Parameters.PLNMethod
- Parameters.SCHMethod
- ParetoDistr
- Pe
- PeList
- PeProvisioner
- PeProvisionerSimple
- PipelineRuntimeVariance
- PlanetLabConstants
- PlanetLabHelper
- PlanetLabRunner
- PlanetLabRunnerConsole
- *PlannerInterface*
- PowerDatacenter
- PowerDatacenterBroker
- PowerDatacenterNonPowerAware
- PowerHost
- PowerHostUtilizationHistory
- *PowerModel*
- PowerModelCubic
- PowerModelLinear
- PowerModelSpecPower
- PowerModelSpecPowerHpProLiantMl110G3PentiumD930
- PowerModelSpecPowerHpProLiantMl110G4Xeon3040
- PowerModelSpecPowerHpProLiantMl110G5Xeon3075
- PowerModelSpecPowerIbmX3250XeonX3470
- PowerModelSpecPowerIbmX3250XeonX3480
- PowerModelSpecPowerIbmX3550XeonX5670
- PowerModelSpecPowerIbmX3550XeonX5675
- PowerModelSqrt
- PowerModelSquare
- PowerVm
- PowerVmAllocationPolicyAbstract
- PowerVmAllocationPolicyMigrationAbstract
- PowerVmAllocationPolicyMigrationInterQuartileRange
- PowerVmAllocationPolicyMigrationLocalRegression
- PowerVmAllocationPolicyMigrationLocalRegressionRobust
- PowerVmAllocationPolicyMigrationMedianAbsoluteDeviation
- PowerVmAllocationPolicyMigrationStaticThreshold
- PowerVmAllocationPolicySimple
- PowerVmList
- PowerVmSelectionPolicy
- PowerVmSelectionPolicyMaximumCorrelation
- PowerVmSelectionPolicyMinimumMigrationTime
- PowerVmSelectionPolicyMinimumUtilization
- PowerVmSelectionPolicyRandomSelection
- Predicate
- PredicateAny
- PredicateFrom
- PredicateNone
- PredicateNotFrom
- PredicateNotType
- PredicateType
- RamProvisioner
- RamProvisionerSimple
- RandomConstants
- RandomHelper
- RandomPlanner
- RandomRunner
- ReclusteringEngine
- ReplicaCatalog
- ReplicaCatalog.FileSystem
- ResCloudlet
- ResCloudletList
- RootSwitch
- RunnerAbstract
- SanStorage
- *SchedulerInterface*
- SimEntity
- SimEvent
- StaticScheduler
- *Storage*
- Switch
- Task
- TaskSet
- TaskStage
- TestBagofTaskApp
- TestExample
- ThrMc
- ThrMc
- ThrMmt
- ThrMmt
- ThrMu
- ThrMu
- ThrRs
- ThrRs
- TopologicalGraph
- TopologicalLink
- TopologicalNode
- UniformDistr
- *UtilizationModel*
- UtilizationModelFull
- UtilizationModelNull
- UtilizationModelPlanetLabInMemory
- UtilizationModelStochastic
- VerticalBalancing
- VerticalClustering
- Vm
- VmAllocationPolicy
- VmAllocationPolicySimple
- VmList
- VmScheduler
- VmSchedulerSpaceShared
- VmSchedulerTimeShared
- VmSchedulerTimeSharedOverSubscription
- VmStateHistoryEntry
- WeibullDistr
- WorkflowApp
- WorkflowEngine
- WorkflowParser
- WorkflowPlanner
- WorkflowScheduler
- WorkflowSimExample1
- WorkflowSimExample2
- WorkflowSimTags
- WorkloadFileReader
- *WorkloadModel*
- ZipfDistr
